# Imbalanced temporal states of cortical blood-oxygen-level-dependent signal variability during rest in episodic migraine

**DOI:** 10.1186/s10194-024-01824-0

**Published:** 2024-07-16

**Authors:** Dániel Veréb, Nikoletta Szabó, Bálint Kincses, Laura Szücs-Bencze, Péter Faragó, Máté Csomós, Szabolcs Antal, Krisztián Kocsis, Bernadett Tuka, Zsigmond Tamás Kincses

**Affiliations:** 1 Department of Radiology, Albert Szent-Györgyi Health Centre, University of Szeged, Semmelweis u. 6, Szeged, 6725 Hungary; 2 Department of Neurology, Albert Szent-Györgyi Health Centre, University of Szeged, Szeged, Hungary; 3 Institute for Diagnostic and Interventional Radiology and Neuroradiology, University Medicine Essen, Essen, Germany; 4 Department of Neurology, Center for Translational Neuro- and Behavioural Sciences, University Medicine Essen, Essen, Germany

**Keywords:** Episodic migraine, Functional MRI, BOLD signal, BOLD variability, Temporal states

## Abstract

**Background:**

Migraine has been associated with functional brain changes including altered connectivity and activity both during and between headache attacks. Recent studies established that the variability of the blood-oxygen-level-dependent (BOLD) signal is an important attribute of brain activity, which has so far been understudied in migraine. In this study, we investigate how time-varying measures of BOLD variability change interictally in episodic migraine patients.

**Methods:**

Two independent resting state functional MRI datasets acquired on 3T (discovery cohort) and 1.5T MRI scanners (replication cohort) including 99 episodic migraine patients (n_3T_ = 42, n_1.5T_=57) and 78 healthy controls (n_3T_ = 46, n_1.5T_=32) were analyzed in this cross-sectional study. A framework using time-varying measures of BOLD variability was applied to derive BOLD variability states. Descriptors of BOLD variability states such as dwell time and fractional occupancy were calculated, then compared between migraine patients and healthy controls using Mann-Whitney U-tests. Spearman’s rank correlation was calculated to test associations with clinical parameters.

**Results:**

Resting-state activity was characterized by states of high and low BOLD signal variability. Migraine patients in the discovery cohort spent more time in the low variability state (mean dwell time: *p* = 0.014, median dwell time: *p* = 0.022, maximum dwell time: *p* = 0.013, fractional occupancy: *p* = 0.013) and less time in the high variability state (mean dwell time: *p* = 0.021, median dwell time: *p* = 0.021, maximum dwell time: *p* = 0.025, fractional occupancy: *p* = 0.013). Higher uptime of the low variability state was associated with greater disability as measured by MIDAS scores (maximum dwell time: *R* = 0.45, *p* = 0.007; fractional occupancy: *R* = 0.36, *p* = 0.035). Similar results were observed in the replication cohort.

**Conclusion:**

Episodic migraine patients spend more time in a state of low BOLD variability during rest in headache-free periods, which is associated with greater disability. BOLD variability states show potential as a replicable functional imaging marker in episodic migraine.

**Supplementary Information:**

The online version contains supplementary material available at 10.1186/s10194-024-01824-0.

## Introduction

Migraine is a common primary headache disorder, which affects the brain on both the structural and functional level [1]. A substantial number of studies investigated migraine-related functional alterations in the brain using fMRI. These studies mainly focused on measures of inter-regional coupling such as functional connectivity, which seems to be altered during and between migraine attacks [2].

Abnormalities of network-level and intra-regional functional activity have also been reported. In a previous study, we found that interictal amplitudes of activity in several resting state networks (e.g., visual, frontoparietal, default mode) were higher in migraine with aura patients [3]. Intra-regional alterations, such as a change in the amplitude of low-frequency fluctuations (ALFF [4]), were also described within regions related to visual processing or pain modulation. Although the biological basis for these alterations is not clear, several hypotheses have been proposed that include altered excitability [5], disbalance of neuromodulatory neurotransmitter levels [6] or the phenomenon of cortical spreading depression [7]. Apart from neuronal sources, other changes in physiological processes might influence properties of the BOLD signal, such as respiratory [8] and cardiac pulsations [9–11] or altered cerebral blood flow [12, 13]. The latter has also been reported in migraine [14]. Recent studies suggest that the variability of the BOLD signal contains information over and above amplitude and has a fundamental role in the functional organization of the brain [15, 16]. While traditional measures of variability (such as variance) incorporate and may relate to amplitude, it also has aspects that pick up on repetitiveness or rigidity in temporal patterns [17]. This dimension of variability in the BOLD signal has so far been understudied in migraine.

Alterations of BOLD variability have been reported during aging [15], and it was also linked to cognitive performance [18]. Altered BOLD variability has been reported in a wide range of neuropsychiatric and metabolic disorders, such as Alzheimer’s disease [19], small vessel disease [13], drug-resistant epilepsy [20], generalized anxiety disorder [21] and chronic kidney disease [22]. Additionally, measures of BOLD variability show promise as biomarkers in psychiatric disorders, where they can be used to monitor treatment response, making them potentially useful in a clinical setting as well [23].

However, an important aspect of BOLD variability has so far been mostly overlooked, especially with regards to the resting state. The BOLD signal, both during rest and task performance, is non-stationary, meaning that its statistical properties (including variance) are not uniform through time [24]. Since functional connectivity between regions also fluctuates in time, and these fluctuations can be categorized into distinct temporal states [25, 26], it is possible that such states also occur in the variability of the BOLD signal. Indeed, several studies reported BOLD variability changes in response to task demands [18]. In the absence of determinable states that conform to e.g., an external task, clustering techniques can be used to derive states, which have been effectively used in estimating resting-state time-varying functional connectivity [25]. Altered brain dynamics during rest have been reported in migraine [27, 28], which might be accompanied by dynamic changes in BOLD signal variability as well.

In this study, we investigate resting-state BOLD signal variability in episodic migraine patients with and without aura during the interictal state. We outline an approach to derive temporal states of BOLD variability, which we hypothesize to be altered in migraine patients. Finally, to address growing concerns about the reproducibility of functional imaging markers in migraine [29, 30], we replicate our results in an independent cohort.

## Materials and methods

### Participants

In this study, we analyzed a discovery dataset consisting of 42 episodic migraine patients and 46 healthy controls, and a previously published replication dataset of 57 episodic migraine patients and 32 healthy controls [27]. All migraine patients have been recruited at the Headache Outpatient Clinic of the Department of Neurology, University of Szeged (between 2018 and 2023 for the discovery dataset, and between 2010 and 2016 for the replication dataset). Patients have been diagnosed with episodic migraine according to the International Headache Society criteria and had no neurological or psychiatric conditions apart from migraine. Healthy controls had no neurological or psychiatric conditions. 16 and 20 migraine patients experienced aura symptoms in the discovery and replication dataset, respectively. The remaining patients reported no experience of aura symptoms. Patients were headache-free at least 48 h before and after the scans. All participants provided their written informed consent according to the Declaration of Helsinki. The local ethics committee of the University of Szeged and the Hungarian Medical Research Council (ETT TUKEB) approved the study (ref. no 057617/2015/OTIG for the discovery cohort and 87/2009 for the replication cohort). The demographic data of the participants can be found in Table 1.

**Table 1:** Demographic data of participants in the analyzed cohorts

	Healthy (discovery cohort)	Migraine (discovery cohort)	Healthy (replication cohort)	Migraine (replication cohort)
n	46	42	32	57
Age (years, mean +/- SD)	25.04 +/- 3.26	29.19 +/- 7.54	35.4 +/- 11.3	34.63 +/- 8.79
Sex (M/F)	21/25	9/33	3/29	6/51
Presence of aura symptoms	-	16	-	20
Disease duration (years, mean +/- SD)	-	11.62 +/- 8.48	-	15.40 +/- 10.21
Attack frequency (attacks/year, mean +/- SD)	-	34.83 +/- 27.50		45.18 +/- 40.02
Allodynia score (median, range)	-	2 (0–12)	-	2 (0–12)
MIDAS score (mean +/- SD)	-	34.41 +/- 29.13	-	-
Interval therapy		4 iprazochrome, 1 beta-blocker, 3 topiramate		5 iprazochrome, 1 amytriptillin, 1 topiramate

### Scanning protocols

Participants included in the discovery dataset were scanned on a 3T GE MR750W Discovery MRI scanner. Structural T1-weighted scans (3D T1-weighted FSPGR-IR sequence, TR: 5.3 ms, TE: 2.1 ms, TI: 450 ms, matrix: 256 × 256, FOV 256 mm × 256 mm, slice thickness: 1 mm, flip angle: 12°, whole brain coverage) and resting-state functional MRI scans (T2*-weighted GE-EPI sequence, TR: 2500 ms, TE: 27 ms, in-plane resolution: 3 mm × 3 mm, FOV: 288 mm x 288 mm, matrix 96 × 96, slice thickness: 3 mm, flip angle: 81°, 44 axial slices providing whole-brain coverage, interleaved acquisition scheme, 168 volumes, 7-minute long scans) were obtained.

For the replication dataset, participants underwent measurements on a 1.5T GE Signa Excite HDxt MRI scanner. Similarly to the discovery dataset, structural T1-weighted scans (3D T1-weighted FSPGR-IR sequence TR: 10.3 ms, TE : 4.2 ms, TI: 450 ms, matrix: 256 × 256, FOV 256 mm × 256 mm, slice thickness: 1 mm, flip angle: 15°, whole brain coverage) and resting-state functional MRI scans (T2*-weighted GE-EPI sequence, TR: 3000 ms, TE: 40 ms, in-plane resolution: 4.7 mm x 4.7 mm, FOV: 300 mm x 300 mm, matrix: 64 × 64, slice thickness: 6 mm, flip angle: 90°, 25 slices providing whole brain coverage, 200 volumes, 10 min-long scans) were acquired.

Participants were asked to lie awake in the scanner with their eyes open and move as little as possible throughout the scanning sessions. We opted for eyes open acquisition as it is associated with a more exteroceptive state, with higher connectivity in networks also affected by migraine [29, 31].

### Image preprocessing

Both datasets were preprocessed with FSL FEAT v.6.0.0 (FSL v5.0.10, [32]). The first 3 and 2 volumes of functional scans in the discovery and replication cohort, respectively (due to repetition time differences), were removed to allow for steady-state magnetization. Motion correction was performed with a rigid-body realignment procedure implemented in FSL MCFLIRT. Non-brain tissue was removed from the images with FSL BET [33]. Afterwards, functional scans underwent slice timing correction and spatial smoothing with a Gaussian kernel of 6 mm full-width-at-half-maximum. The alignment of functional scans to MNI template space was performed using a two-stage registration process consisting of boundary-based registration to structural scans followed by non-linear spatial normalization to standard 2 mm MNI-space using FSL FNIRT. Further motion correction was applied using separate strategies in the two datasets. In the discovery dataset, ICA-AROMA was applied to remove motion-related artifacts [34]. The 24-parameter head motion model was used to correct for motion artifacts in the replication dataset [35]. The reason for this distinction is two-fold. First, although ICA-AROMA has been tested on 1.5T fMRI data, more detailed benchmarking has been performed on standard-acquisition 3T data [36, 37]. Second, the choice of motion correction strategies in the preprocessing pipeline influences results [37], therefore we considered it important to see if our results were replicable with different motion correction strategies. Following motion correction, nuisance regression was performed to remove white matter and CSF signals and a high-pass filter was applied to resulting timeseries with a 0.008 Hz cutoff. To see if other physiological components of the BOLD signal (such as CSF fluctuations arising from cardiac pulsations) influence our results, we repeated the analysis without correcting for white matter and CSF signals (see Supplementary material).

### Calculation of BOLD signal variability

**Fig. 1:**
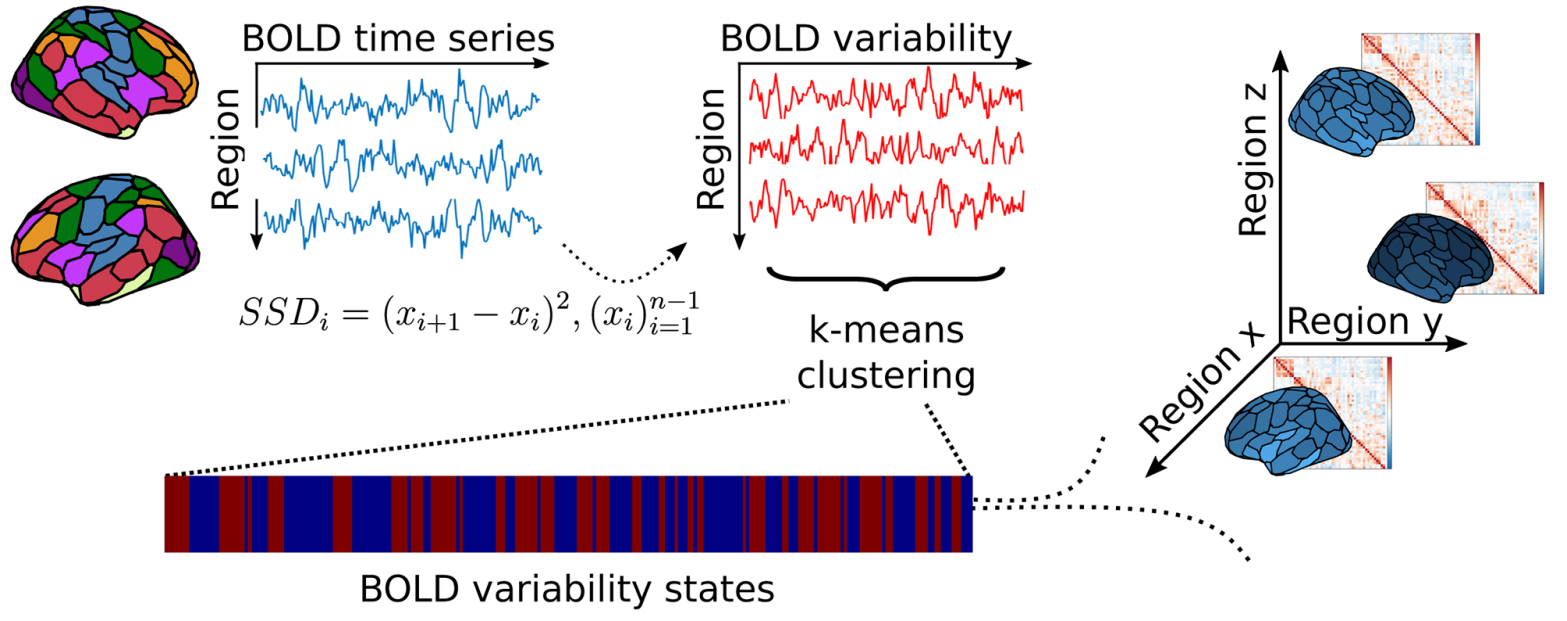
Schematic depiction of the analysis workflow. Average time series underlying regions from the 100-parcel Schaefer atlas were extracted from preprocessed functional scans. Frame wise BOLD SSD was calculated as per the equation in the figure, where *i* denotes the time point, *x* denotes normalized BOLD activity at the given time point, and *n* represents the total number of scans. After calculating frame wise BOLD SSD, k-means clustering was applied to SSD time series to derive temporal clusters (states) of BOLD variability. These states can be represented in a coordinate system plot (shown on the right), where axes represent the momentary BOLD signal change in different regions (denoted by regions x, y, z here for simplicity). Each point in this coordinate system represents a state characterized by a distinct multi-regional pattern of BOLD variability, creating a state space traversed by the brain throughout the scanning process. Abbreviations: BOLD: blood oxygen level dependent; SSD: successive squared difference

The 100-parcel resolution Schaefer-atlas was used to parcellate functional scans [38]. The mean timeseries of voxels underlying each parcel was extracted, then demeaned and normalized to unit standard deviation. The latter analysis step was included to ensure that differences in BOLD variability were not driven by differences in BOLD signal amplitude reported by several studies in migraine ( [3, 4]). BOLD variability was calculated as the successive squared difference (SSD) of signal intensity at each timepoint [39]. To make temporal clustering (as described in the next section) feasible, we omitted the temporal averaging step in the denominator for that specific analysis, which resulted in time series equivalent to the squared first-order temporal derivative of the BOLD signal (Fig. 1). To facilitate readability, we will refer to SSD as BOLD variability for the rest of the manuscript.

### Deriving temporal states of BOLD signal variability

Timeseries of BOLD variability were arranged in a timepoint by region matrix for each subject. To find different states of BOLD variability patterns, k-means clustering was applied to the temporally concatenated variability matrices of the discovery and replication cohort separately in the temporal domain following previous literature on time-varying functional connectivity and brain co-activation states [26]. The k-means clustering algorithm was run for 2 to 10 clusters, and the Calinski-Harabasz clusterability index was calculated to find the optimal number of clusters. Several descriptors were calculated for each state, such as mean, median and maximum dwell time (average, median and maximal time the given participant spent in a certain state) and fractional occupancy (the ratio of time spent in a certain state and the full duration of the scan). Furthermore, we conducted a functional connectivity analysis to check whether BOLD variability states were associated with different functional connectivity patterns. Similarly to the variability analysis, average regional time series from the 100-parcel Schaefer-atlas were extracted. We calculated inter-regional functional connectivity (FC) as the Pearson’s correlation between regional BOLD timeseries during different BOLD variability states. Each region was assigned a network membership as implemented in the Schaefer-atlas (which is aligned to the 7 canonical resting state networks established on the Yeo-parcellation [38, 40]). Finally, we compared FC between states in the healthy group via a non-parametric permutation test as implemented in FSL randomise [41].

### Calculating activation and deactivation ratios

BOLD activation and deactivation events are associated with distinct changes in neurotransmitter levels, and point to changes in excitatory/inhibitory balance [42]. To better understand how alterations in BOLD variability are associated with the ratio of activation and deactivation events in the analyzed cohorts, we performed the following analysis. It has been shown that large amplitude BOLD signal changes denote activation events during the resting state, from which resting state co-activation patterns can be extracted that resemble canonical resting state networks [43]. We hypothesized that, based on these findings, when the BOLD signal drops below a certain threshold, deactivation occurs during the resting state. To see how much time participants spend in “active” and “deactive” conditions, we calculated the number of time points when the normalized BOLD time series exceed + 1 and − 1 standard deviation (SD) for each participant. These occurrences were then normalized by the total number of time points in the BOLD time series to obtain activation and deactivation ratios. We analyzed average activation and deactivation ratios in each of the 7 resting state networks as defined in the Schaefer-atlas because of regional differences in neurotransmitter levels and BOLD properties [42].

### Statistical analysis

State descriptors were corrected for linear and quadratic age effects, linear sex effects, and compared between groups with Mann-Whitney U-tests. Correction for multiple comparisons was performed using false discovery rate correction via the Benjamini-Hochberg method. A second comparison was run between migraine patients with and without aura. The relationship between state descriptors and clinical parameters, as well as state descriptors and activation-deactivation ratios, was assessed with the partial Spearman’s rank correlation coefficient while controlling for age, sex, disease duration and the presence of aura symptoms.

## Results

### Resting state BOLD activity is organized into alternating low and high variability states

**Fig. 2:**
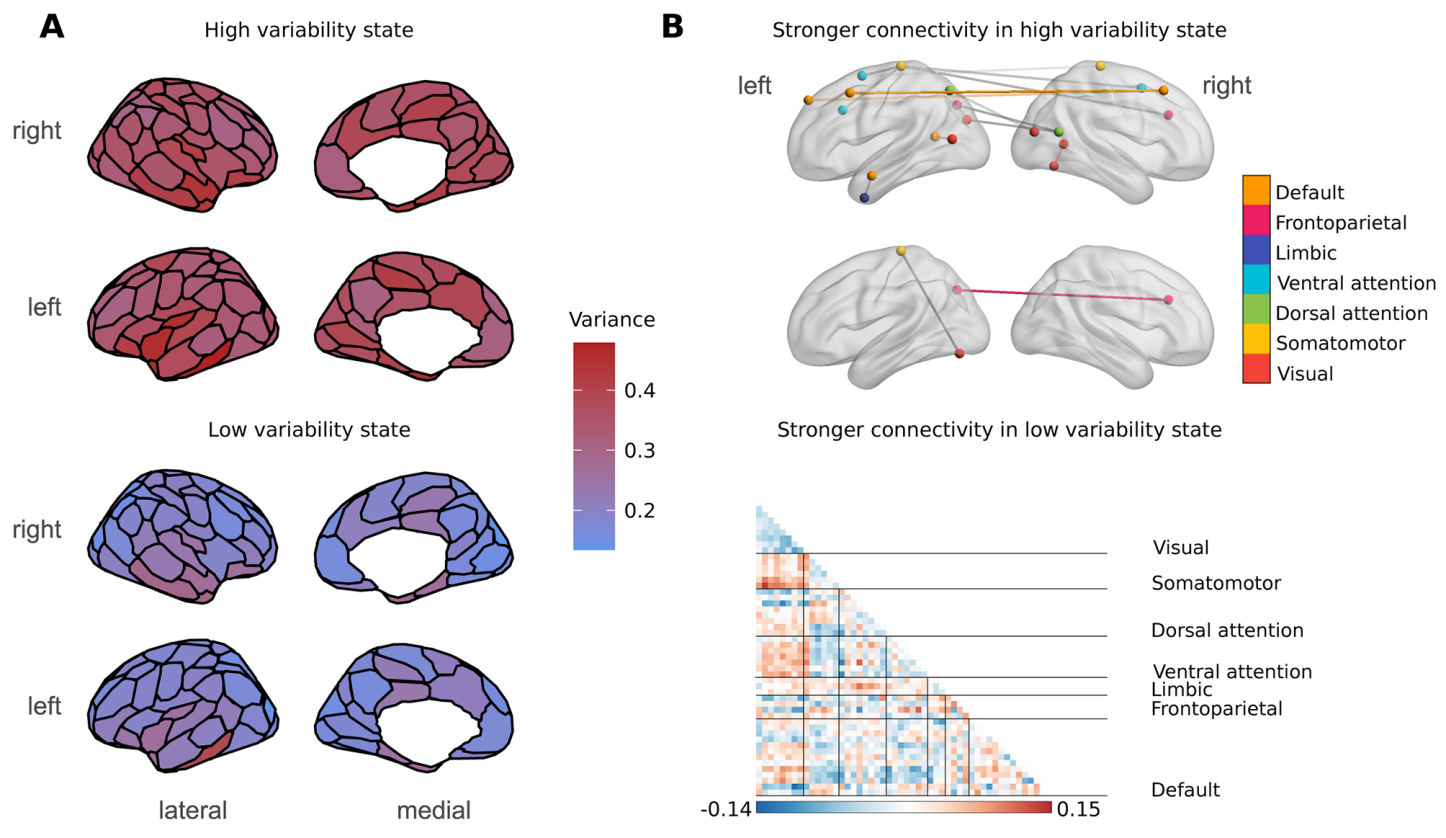
Regional distribution of BOLD variability during high and low variability states and association with functional connectivity. Mean successive squared difference of BOLD activity in the healthy group of the discovery dataset during high and low variability states was overlaid on corresponding regions from the 100-parcel Schaefer atlas (panel **A**). The high variability state was associated with stronger within-network connectivity in the default-mode network and stronger between-network connectivity between regions of the default-mode, ventral/dorsal attention, somatomotor and visual networks. The low variability state was associated with stronger within-network connectivity in the frontoparietal/executive network and stronger connectivity between regions of the somatomotor and visual networks (panel **B**). Edges are colored according to network membership and between-network edges are colored in gray. The matrix plot shows region wise differences of functional connectivity between the high and low variability states, in a way that negative values (in blue) show greater connectivity in the low variable state, and positive values (red-brown) show greater values in the high variability state

After concatenating subject-wise BOLD variability time series, k-means clustering partitioned time series into 2 states, a low and a high variability state (Fig. 2A). This was consistent across the discovery and replication datasets. The high variability state was associated with stronger within-network connectivity in the default-mode network and stronger between-network connectivity between regions of the default-mode, ventral/dorsal attention, somatomotor and visual networks. The low variability state was associated with stronger within-network connectivity in the frontoparietal/executive network and stronger connectivity between regions of the somatomotor and visual networks (Fig. 2B).

### Migraine patients spend more time in the low variability state

Regarding BOLD mean variability, we observed no differences between patients and healthy controls in either of the two datasets.

**Fig. 3:**
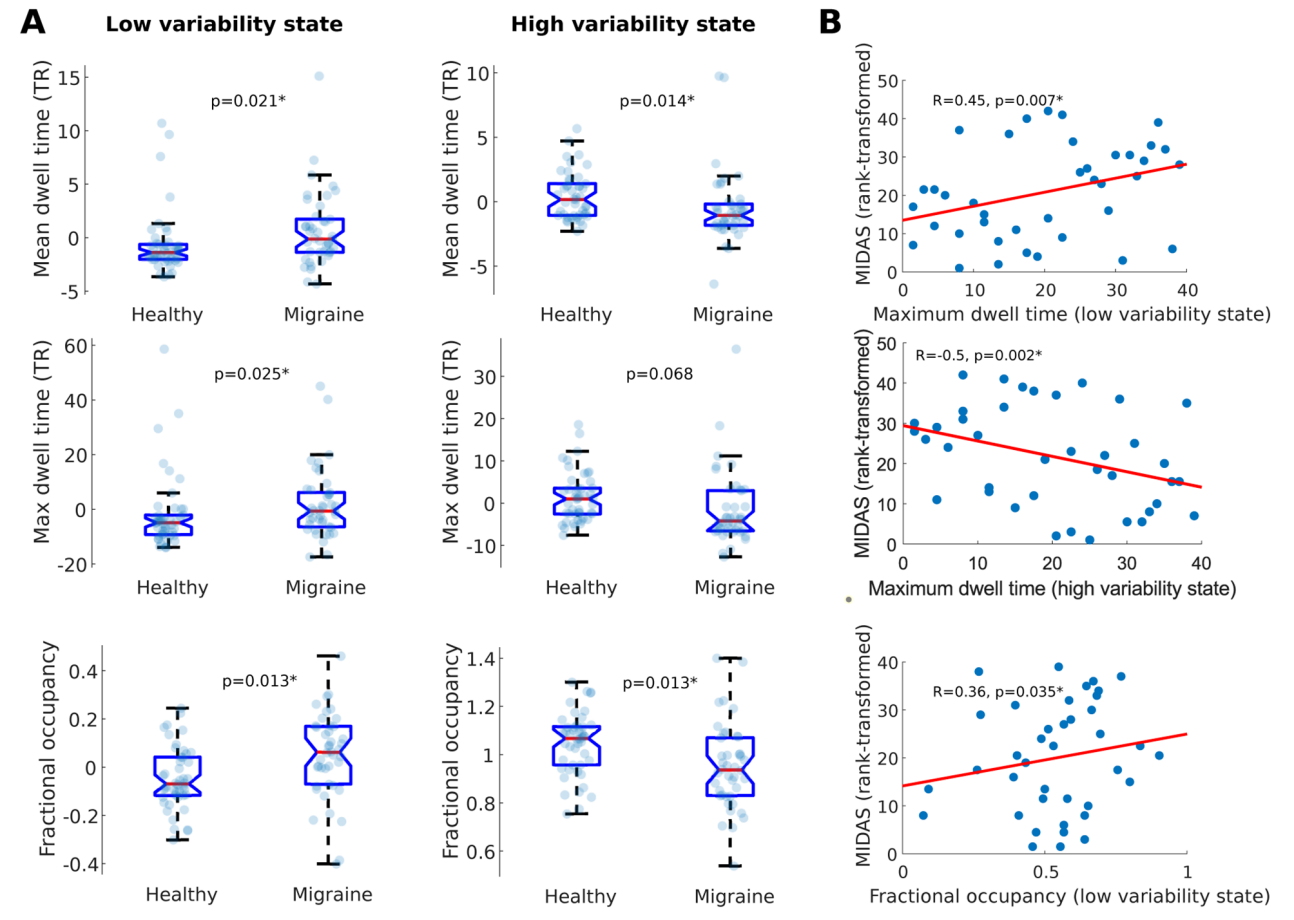
Comparison of BOLD variability state descriptors between groups and associations with disability. Boxplots in panel **A** denote the distribution of state descriptor values in the healthy and migraine groups from the 3T dataset during low and high BOLD variability states. State descriptor values depicted here are adjusted for age and sex. Red lines denote the median, the box borders denote 25th and 75th percentiles, whereas whiskers denote 10th and 90th percentiles. Scatter plots in panel **B** depict the relationship between rank-transformed MIDAS scores and different state descriptors in the migraine group, with a least squares line superimposed in red. Significant differences and correlations are denoted with an asterisk

Patients with migraine in the discovery dataset spent more time on average in the low variability state (mean dwell time: p_FDR_=0.014, median dwell time: p_FDR_=0.022, maximum dwell time: p_FDR_=0.013, fractional occupancy: p_FDR_=0.013), and less time in the high variability state (mean dwell time: p_FDR_=0.021, median dwell time: p_FDR_=0.021, maximum dwell time: p_FDR_=0.025, fractional occupancy: p_FDR_=0.013) (Fig. 3A). These effects were independent of age or biological sex. Retaining CSF and white matter signal in the time series diminished the sensitivity of our measures to group membership (see Supplementary material).

Consistently with these results, migraine patients in the replication dataset also spent more time in the low variability state (maximum dwell time: p_FDR_=0.038, there was also a trend of increased fractional occupancy (p_FDR_=0.078)) and less time in the high variability state (median dwell time: p_FDR_=0.034; with a trend of reduced mean and maximum dwell time (p_FDR_=0.078 and p_FDR_=0.068 respectively)), controlling for age and sex effects (Figure S1).

By pooling the discovery and replication datasets, we observed stronger group differences. Migraine patients in the pooled dataset spent more time on average in the low variability state (mean dwell time: p_FDR_=0.002, maximum dwell time: p_FDR_=0.001, fractional occupancy: p_FDR_=0.001) and less time in the high variability state (mean dwell time: p_FDR_=0.002, median dwell time: p_FDR_=0.002, maximum dwell time: p_FDR_=0.001, fractional occupancy: p_FDR_=0.001), accounting for the effects of age and sex.

### Higher uptime of the low variability state is associated with greater disability in migraine patients

Irrespective of age, sex, disease duration and presence of aura symptoms, higher uptime of the low variability state was associated with higher disability according to the MIDAS questionnaire (maximum dwell time: *R* = 0.45, *p* = 0.007; fractional occupancy: *R* = 0.36, *p* = 0.035) and higher average pain severity (on the visual analogue scale) during headaches (fractional occupancy: *R* = 0.34, *p* = 0.039), whereas higher uptime of the high variability state was associated with lower disability according to the MIDAS questionnaire (maximum dwell time: *R*=-0.51, *p* = 0.002)(Fig. 3B). When we explicitly tested for differences between patients who experienced aura symptoms and patients who did not, we observed no significant results.

In the replication dataset, significant associations between state descriptors and clinical parameters were only observed in the migraine with aura group. Higher uptime of the low variability state was associated with greater attack frequency (median dwell time: *R* = 0.49, *p* = 0.037) and disease duration (median dwell time: *R* = 0.48, *p* = 0.043) and higher uptime of the high variability state was associated with lower disease duration (median dwell time: *R*=-0.49, *p* = 0.039), when controlling for age and sex only. With the inclusion of disease duration and presence of aura symptoms as confounds, we observed no significant correlation between clinical parameters and state descriptors in the replication cohort.

When pooling the two datasets, we observed no significant correlation between state descriptors and clinical parameters available in both groups (attack frequency, total attack number, attack duration, allodynia scores, VAS of pain during headaches) when controlling for age, sex, disease duration and presence of aura symptoms. Looking separately at migraine patients with and without aura and controlling for age and sex effects, higher uptime of the low variability state was associated with higher VAS scores during headaches (*R* = 0.32, *p* = 0.013) in the migraine without aura group.

### Higher uptime of the low variability state is associated with fewer deactivation events in migraine

**Fig. 4:**
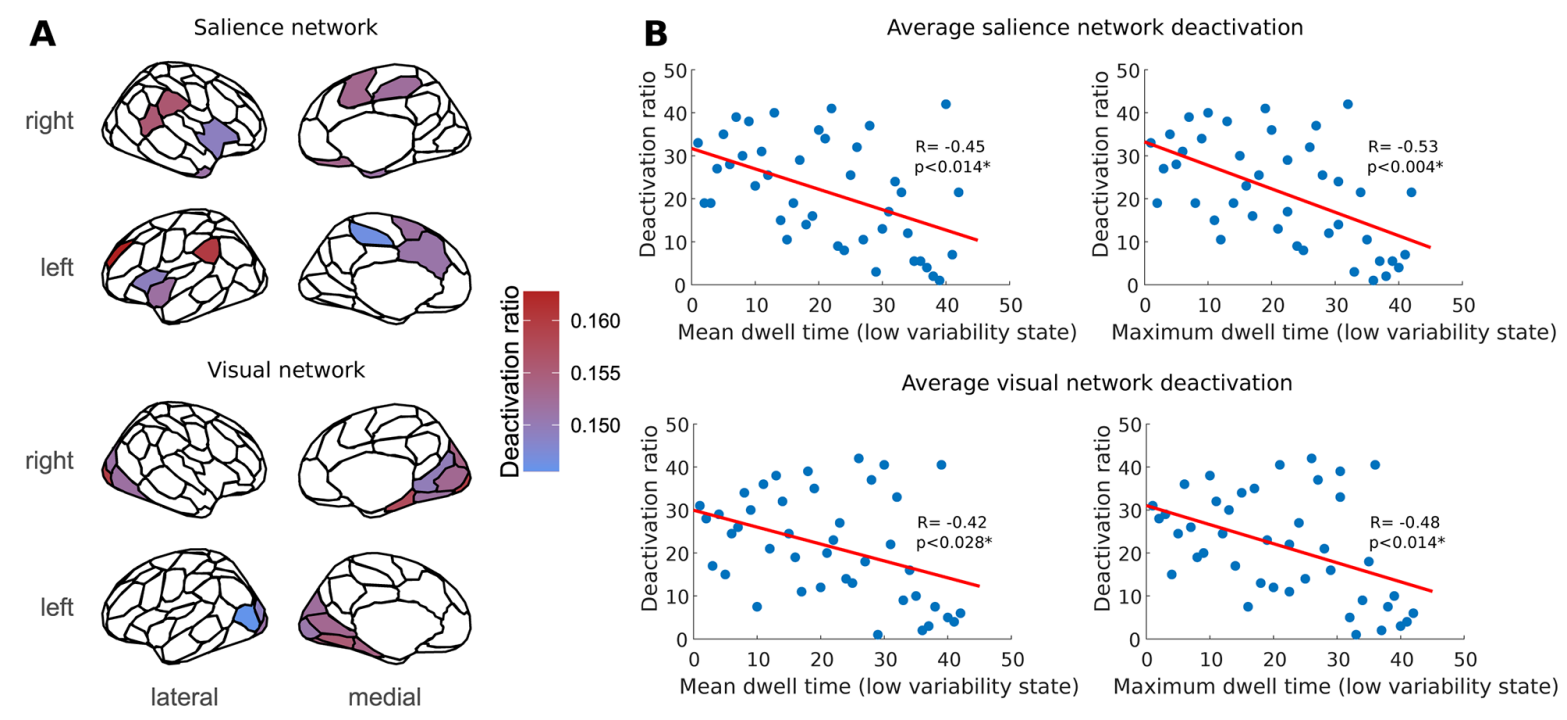
Higher uptime of the low BOLD variability state is associated with fewer deactivation events in migraine. Region wise deactivation ratios in the visual and salience networks in the migraine group of the discovery dataset during high and low variability states was overlaid on corresponding regions from the 100-parcel Schaefer atlas (panel **A**). Scatter plots in panel **B** depict the relationship between rank-transformed average deactivation ratios in the visual and salience networks and different state descriptors in the migraine group, with a least squares line superimposed in red. Significant differences and correlations are denoted with an asterisk

Higher uptime of the low variability state in the 3T migraine group was associated with a decreased deactivation ratio in the visual network (mean dwell time: *R* = -0.42, p_FDR_ = 0.028; maximum dwell time: *R* = -0.48, p_FDR_ = 0.014) and the salience network (mean dwell time: *R* = -0.45, p_FDR_ = 0.014; median dwell time: *R* = -0.40, p_FDR_ = 0.028; maximum dwell time: *R* = -0.53, p_FDR_ = 0.004; fractional occupancy: *R* = -0.36, p_FDR_ = 0.044) irrespective of age, sex and aura status (see Fig. 4).

We observed a similar effect regarding the visual network in the 1.5T dataset (median dwell time: *R* = -0.37, p_FDR_ = 0.04).

When pooling the discovery and replication datasets together, these associations remained significant in the visual (mean dwell time: *R* = -0.31, p_FDR_ = 0.013; median dwell time: *R* = -0.29, p_FDR_ = 0.028; maximum dwell time: *R* = -0.28, p_FDR_ = 0.013; fractional occupancy: *R* = -0.25, p_FDR_ = 0.028) and salience networks (mean dwell time: *R* = -0.34, p_FDR_ = 0.002; median dwell time: *R* = -0.26, p_FDR_ = 0.018; maximum dwell time: *R* = -0.35, p_FDR_ = 0.002; fractional occupancy: *R* = -0.26, p_FDR_ = 0.018), and we observed additional significant associations in the dorsal attention network (mean dwell time: *R* = -0.37, p_FDR_ = 0.001; median dwell time: *R* = -0.27, p_FDR_ = 0.014; maximum dwell time: *R* = -0.39, p_FDR_ = 0.001; fractional occupancy: *R* = -0.30, p_FDR_ = 0.007).

We found no significant associations between state descriptors and activation ratios, nor activation/deactivation ratios. Activation and deactivation ratios did not differ between healthy and migraine groups in any resting state networks.

## Discussion

In this study, we investigated alterations of the temporal aspects of BOLD signal variability in episodic migraine patients. We found that during rest, states of low and high BOLD signal variability alternate, switching every 10–15 s on average. Migraine patients, when headache-free, spent more time in the low variability state, which was associated with greater disability. We obtained similar results in an independent cohort of migraine patients and healthy controls even with different preprocessing pipelines. These findings provide important complementary information about altered brain dynamics in migraine patients and open up new avenues for the discovery of disease-specific imaging markers.

Alterations of the BOLD signal in migraine have been a subject of investigation for years. Some studies found differences of the hemodynamic response function itself which underlies the observed BOLD signal [44]. Other studies found that the amplitude of BOLD fluctuations is generally altered in migraine patients, both on the intra-regional and network levels [3, 4]. Our current results provide complementary information to the findings of these studies, suggesting that migraine patients spend more time in a state where the variability of the BOLD signal is lower. By normalizing time series to unit standard deviation, we remove the confound of possible amplitude differences, and the resulting variability reflects the degree to which timeseries are dominated by recurrent patterns or motifs or, alternatively, by elongated periods of activation or deactivation [17]. Therefore, our current results can be interpreted in a way that BOLD activity during the low variability state assumes values from a more limited range of possible values, resulting in the decrease of the dynamic range of the BOLD signal. We demonstrated that higher occurrences of such a low variability state are associated with greater disability.

A possible underlying mechanism for these findings might stem from the imbalance of excitatory and inhibitory neurotransmitter levels in migraine. While reports of alterations in the cerebral levels of glutamate and GABA in migraine are conflicting, several studies reported increases of regional glutamate and decreases of regional GABA levels [45]. Since these neurotransmitters are associated with BOLD activation and deactivation, respectively [42, 46], this hypothetically provides grounds for altered activation and deactivation ratios in migraine patients that could result in lower variability of the BOLD signal during these periods. To test this hypothesis, we performed an analysis where we obtained the ratio of activation and deactivation events during the scan. We observed that increased uptime of the low variability state in migraine patients was associated with fewer deactivation events in the visual, salience and, regarding pooled datasets, the dorsal attention networks. These networks have been implicated in migraine and have important roles in pain processing and visuospatial ability [47, 48]. BOLD deactivation events in the visual network were shown to be associated with reductions in glutamate, lactate and GABA concentrations, as well as reduced glucose and increased glutathione levels [46, 49]. More sparse visual deactivation events associated with a higher uptime of the low BOLD variability state are therefore in line with previous findings of increased glutamate and lactate levels in the occipital cortex of migraine patients [45]. Our research group also reported more unstable connections within the salience network and impaired information flow between salience and dorsal attention networks [27].

Importantly, alterations of BOLD variability in migraine might be connected to other physiological sources that influence the BOLD signal, such as respiratory or cardiac pulsations and changes in cerebral blood flow and volume [8, 10–14]. When repeating the analysis without regressing out CSF and white matter signals, however, we found that group differences were diminished. These results emphasize the importance of nuisance regression and suggest that the increased uptime of low BOLD variability states in migraine patients comes at least in part from changes in brain function [50].

Nevertheless, more research is needed on the biological basis of our findings. Future studies might link the higher uptime of low BOLD variability states directly to cerebral neurotransmitter levels using magnetic resonance spectroscopy or could investigate wave-like phenomena associated with CSD during low BOLD variability states. Although not without technical challenges, assessing BOLD variability during migraine attacks could also help in establishing the mechanisms underlying these alterations.

Interestingly, we did not find differences of mean BOLD variability between the healthy and migraine cohorts. This points to a potential use of time-varying methods in assessing BOLD variability during rest. Task-based studies have shown that BOLD variability is different during task performance and task free periods [18, 23]. Our results suggest that periods of increased and decreased BOLD variability also alternate during rest. We additionally found differences of functional connectivity between high and low BOLD variability states. High BOLD variability was associated with stronger between-network connectivity and stronger connectivity within the default mode network, while connectivity between motor and visual areas, as well as within-network connectivity of the frontoparietal control network was stronger in lower BOLD variability states. These results underline the role of BOLD variability in the organization of functional brain networks [17], and provide new avenues for investigating alterations of functional connectivity in migraine.

A strength of our study is that we obtained similar results in two independent cohorts with slight differences in the preprocessing pipeline. Although we observed fewer group differences in the replication dataset, this could be due to the lower signal-to-noise ratio of the 1.5T acquisition, suggesting that future studies should focus on 3T acquisition. Replicability of results is a growing concern in the migraine neuroimaging literature and presents a challenge in both study design (heterogeneity of clinical manifestations, high co-morbidity with other headache disorders, the paroxysmal nature of migraine, effect of medications etc.) and analysis strategies (harmonization of scanning protocols, motion correction strategies). Although we took a step towards more replicable results on the single-center level, a more optimal solution would be the pooling of data between headache centers and the establishment of larger multicenter databases, which remains a challenge for the field.

This study has some limitations. The current approach of deriving states of BOLD variability requires a control group and is calculated on the population level, which, although it results in individual indices, is not easily applicable in a clinical setting. Furthermore, even though results were similar between 3T and 1.5T datasets, more studies are needed for the validation of BOLD variability differences in migraine.

## Conclusions

In this paper, we present an approach for deriving temporal states of BOLD signal variability during rest. Using this approach, we demonstrated in two independent cohorts that migraine patients spend more time in a low variability state, which was associated with greater disability. Our results provide new avenues for functional imaging markers in migraine and can be applied to other disorders as well.

### Electronic supplementary material

Below is the link to the electronic supplementary material.

Supplementary Material 1

## Data Availability

The datasets analyzed during the current study are available from the corresponding author on reasonable request after consideration by the local ethics committee.
