# Supplementary material for: Imbalanced temporal states of cortical blood-oxygen-level-dependent signal variability during rest in episodic migraine
Source: J Headache Pain. 2024 Jul 16;25(1):114. doi: 10.1186/s10194-024-01824-0 (PMC11251240; doi:10.1186/s10194-024-01824-0)
Supplement: Supplementary file 1 — Supplementary Material 1 [file 10194_2024_1824_MOESM1_ESM.docx]

# Supplementary material


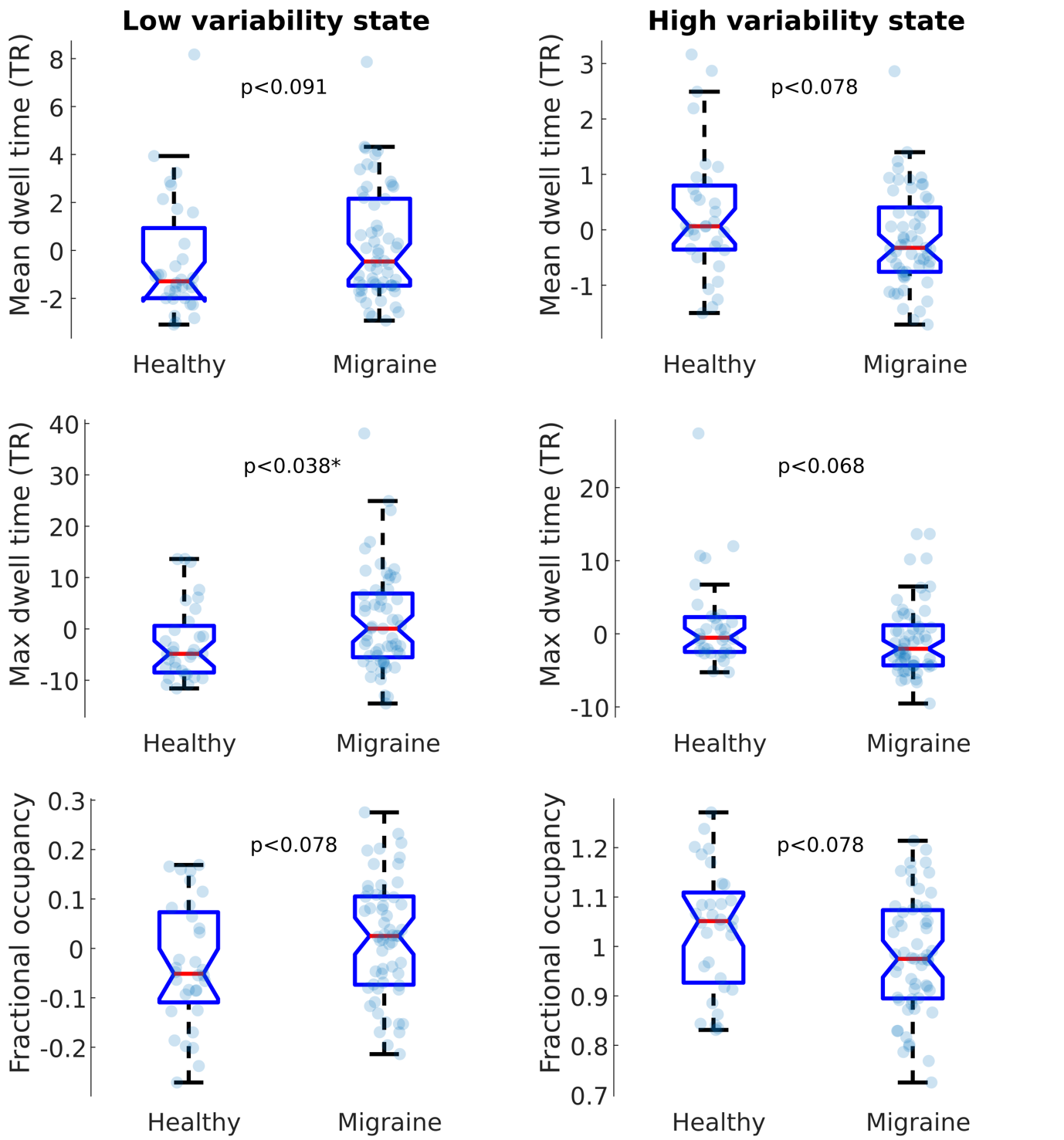


**Figure S1.** **Comparison of BOLD variability state descriptors between groups in the replication dataset.** Boxplots denote the distribution of state descriptor values in the healthy and migraine groups from the 1.5T dataset during low and high BOLD variability states. Significant differences and correlations are denoted with an asterisk.

## Group differences of state descriptors using time series without nuisance regression

When using regional time series for BOLD variability estimation without regressing out CSF and white matter signals, we observed alternating high and low variability states similarly to the main analysis. There was a trend of less time spent in the high variability state in the migraine group (mean and maximum dwell time in the high variability state: uncorrected p=0.02 and p=0.03, respectively). Other state descriptors derived this way did not show differences between the migraine and healthy groups (mean dwell time: p=0.27 low variability state; median dwell time: p=0.24 low variability state, p=0.05 high variability state; maximum dwell time: p=0.80 low variability state; fractional occupancy: p=0.16).
